# Seroprevalence of SARS-CoV-2 infection in the Tyrolean district of Schwaz at the time of the rapid mass vaccination in March 2021 following B.1.351-variant outbreak

**DOI:** 10.3389/fpubh.2022.989337

**Published:** 2022-09-09

**Authors:** Peter Willeit, Janine Kimpel, Hannes Winner, Teresa Harthaller, Helena Schäfer, David Bante, Barbara Falkensammer, Annika Rössler, Lydia Riepler, Cornelia Ower, Magdalena Sacher, Dorothee von Laer, Wegene Borena

**Affiliations:** ^1^Clinical Epidemiology Team, Medical University of Innsbruck, Innsbruck, Austria; ^2^Department of Public Health and Primary Care, University of Cambridge, Cambridge, United Kingdom; ^3^Department of Hygiene, Microbiology and Public Health, Institute of Virology, Medical University of Innsbruck, Innsbruck, Austria; ^4^Department of Economics, University of Salzburg, Salzburg, Austria; ^5^Department of Surgery, University Hospital of Trauma Surgery, Medical University of Innsbruck, Innsbruck, Austria; ^6^Department of Visceral, Transplant and Thoracic Surgery, Center of Operative Medicine, Medical University of Innsbruck, Innsbruck, Austria

**Keywords:** seroprevalence, SARS-CoV-2, vaccination, Comirnaty, undocumented infection, seropositivity, anti-N, beta

## Abstract

In order to curb the rapid dissemination of the B.1.351 variant of SARS-CoV-2 in the district of Schwaz and beyond, the EU allocated additional vaccine doses at the beginning of March 2021 to implement a rapid mass vaccination of the population (16+). The aim of our study was to determine the seroprevalence of SARS-CoV-2 among the adult population in the district of Schwaz at the time of the implementation. Data on previous history of infections, symptoms and immunization status were collected using a structured questionnaire. Blood samples were used to determine SARS-CoV-2 specific anti-spike, anti-nucleocapsid and neutralizing antibodies. We recruited 2,474 individuals with a median age (IQR) of 42 (31–54) years. Using the official data on distribution of age and sex, we found a standardized prevalence of undocumented infections at 15.0% (95% CI: 13.2–16.7). Taken together with the officially documented infections, we estimated that 24.0% (95% CI: 22.5–25.6) of the adult population had prior SARS-CoV-2 infection. Hence, the proportion of undocumented infections identified by our study was 55.8% (95% CI: 52.7–58.5). With a vaccination coverage of 10% among the adults population at that time, we imply that a minimum of two-thirds of the target popuation was susceptible to the circulating threat when this unique campaign started.

## Introduction

As global efforts are in progress to cope with the uncontrolled transmission of SARS-CoV-2 infection, new variants surface as obstacles against the process of containment. The variant B.1.351, detected for the first time in South Africa in October 2020 (1), was found to have impaired neutralization by convalescent plasma from the wild type infection. A potential dominance of such immune escape variants may pose a serious threat to real world vaccine effectiveness.

Almost simultaneously with the introduction of the first vaccines at the beginning of the year 2021, Europe notified increased circulation of the variant B.1.351 (2).

Having reported over 300 cases of infections with this variant of concern (VOC) at that time, Austria came into focus as a hotspot (3, 4). Almost all reported cases originated from the district of Schwaz in the western part of the country. In order to curb the spread of this variant, the European Union chose the district to serve as a model region and provided Austria with 100,000 doses of the BNT162b2 (Comirnaty) vaccine by BioNTech/Pfizer for the immediate immunization of 50,000 adults living in this district. According to official reports, approximately 41,700 (61% of the adult population) received both doses of the vaccine as part of this immunization programme (4, 5).

Vaccine donors, regional or national health authorities, vaccine policy makers, other relevant institutions or even the general public may legitimately wonder what proportion of the community had already been exposed to the virus or what proportion was completely immuno-naïve as this unique immunization campaign started. The results may be used as baseline information in evaluating the performance of the mass immunization in achieving the goal it was aimed for (6). Although for this purpose data from the official registry of SARS-CoV-2 infections may be utilized, it is highly likely that a non-negligible proportion of the population might have gone through an infection that has remained undetected and unreported (7–10). Anti-nucleocapsid antibodies may help identify, irrespective of vaccination status, subjects with prior infections which were not detected by the conventional confirmatory tests.

With this study, we aimed to determine the prevalence SARS-CoV-2 infection status through the use of serological assays in the district of Schwaz at the time of the mass vaccination.

## Materials and methods

### Study population

The study was conducted in March 2021. All adult residents of the district Schwaz in Tyrol, aged 18 years and above (*n* = 68,896), were invited through the local county office and local media. Consenting participants were asked to fill out a short questionnaire on sociodemographic aspects as well as their history of SARS-CoV-2 infection, history of hospitalization and the status of vaccination. Blood samples (EDTA) collected at the study site were used for the determination of SARS-CoV-2-specific antibodies targeting the spike (S) and nucleocapsid (N) proteins.

### SARS-CoV-2 antibody test

All samples were tested using the Chemiluminescent-based immunoassay—SARS-CoV-2 IgG II Quant (Abbott, Illinois, USA). The assay detects antibodies directed against S protein and was performed on the ARCHITECT i2000SR platform. Using standards of various concentrations, results were provided in a quantitative manner as binding antibody units per milliliter (BAU/ml). According to the manufacturer, the cutoff for positivity was defined to be >7.1 BAU/ml. All samples were further analyzed using a second serological assay—the Elecsys Anti-SARS-CoV-2 (Roche Diagnostics, Indianapolis, USA)—which detected antibodies against the N protein of SARS-CoV-2. Detection of anti-N immunoglobulin (anti-N Ig) has the additional value of differentiating between post-infection and post-vaccination antibody positivity. Individuals with positive anti-N Ig were further tested for the presence of neutralizing antibodies using an in-house pseudovirus-based assay (pVNT) as described previously (11, 12). In short, replication defective vesicular stomatitis virus (VSVΔG-GFP) pseudotyped with Wuhan-1 spike protein was used to infect susceptible cells (293T-ACE2) after pre-incubation with participant's plasma in serial four-fold dilutions. Cells infected with the pseudovirus expressed GFP. This signal was quantified approximately 16 hours after infection using a spot reader (ImmunoSpot® S5 analyzer). Continuous titers that resulted in 50% reduction of GFP expression (50% inhibition titer) as compared to virus-only wells were determined using a non-linear regression method as described before (13). Titers ≥1:16 (continuous titer of ≥16) were considered positive.

### Data analysis

Our study population showed a slight predominance of women and subjects between 25 to 55 years of age as compared to the general (source) population (Supplementary Figure 1). In order to account for these discrepancies, we estimated the overall prevalence through age and sex standardization. To counteract a potential selection-bias of the study (i.e., more subjects with a history or suspicion of previous SARS-CoV-2 infection preferentially willing to participate), we estimated the serology-derived proportion of unreported infections and interpreted the result in combination with data from officially reported cases. For this purpose, we excluded subjects who gave a history of prior infection in the questionnaire and considered anti-N positivity among the rest of the study participants to represent the proportion of undocumented infections. Through direct age standardization using the official census data of the district (14), we estimated the seroprevalence in the adult population of Schwaz (source population). We defined the true cumulative incidence at the time of data collection to be the sum of the estimated undocumented infections and the officially reported daily numbers stratified by age and sex kindly provided by the Austrian Agency for Health and Food Safety (Dr. Daniela Schmid, AGES).

We used student's t-test or ANOVA to test for a difference in quantitative variables across groups. We applied non-parametric tests (Mann-Whitney U test and Kruskal-Wallis) for variables not fulfilling the criteria of normality. For variables of categorical nature, we used χ^2^-test (Fisher's exact where appropriate). For the main analysis of seroprevalence of unreported infections using Roche anti-N Ig we also provided a Rogan-Gladen correction for an imperfect diagnostic test. 95% CIs were calculated using the Clopper-Pearson exact method (15). The level of significance was set at 5% using two-sided tests where applicable.

### Ethical clearance

The study was approved by Ethics Committee of the Medical University of Innsbruck (EK Nr:1093/2021).

## Results

### Questionnaire data

As shown in Table 1, the total number of participants added up to 2,474 adults (*n* = 1,028 males and *n* = 1,446 females) between 18 and 89 years of age. While 593 participants (24%, 95% CI: 22.4–25.6) reported to have had a PCR (*n* = 591) or antigen (*n* = 2) confirmed SARS-CoV-2 infection, 94 (3.8%, 95% CI: 3.1–4.5) reported a previous infection based on a routine antibody test. Only 15 participants (2.1%) reported to have been hospitalized due to SARS-CoV-2 infection. Around 15% of subjects with a previous infection reported to have had no symptoms. Among participants with a history of infection and reporting symptoms (*n* = 578), the majority (52.8%) had only mild symptoms without being bedridden. Although symptom reporting was more common among females than males [OR, (95% CI) = 2.03 (1.34–3.14)], significantly more males reported to have been hospitalized [OR, (95% CI) = 3.04 (1.03–8.99)]. A total of 1,948 participants (79.2%) had received at least one dose of an mRNA or a vector vaccine approved in Europe at the time of the study (a single individual reported to have received BBIBP-CorV, Sinopharm). The majority (92%) of the vaccinated subjects had received Comirnaty—as part of the mass vaccination with a median (IQR) of 9 (7–10) days prior to the study—followed by Vaxzevria (ChAdOx1, AstraZeneca) (7.6%). No significant difference was observed in the vaccination status across sexes.

**Table 1:** Baseline characteristics of study participants (n = 2,474).

**Participants**	**Total (*n* = 2,474)**	**Male (*n* = 1,028)**	**female (*n =* 1446)**	p-value^*^	**Missing (n)**
**Age, years**					0
Mean (SD)	43.2 (14.3)	44.3 (14.3)	42,4 (14.2)	**0.001**	
Median IQR	42 (31-54)	44 (33-55)	42 (31-53.3)		
Range	18-86	18-82	18-86		
**History of previous infection (reported) (n,%)**				0.49	10
PCR-based	591 (23.9)	236 (23.3)	355 (24.6)		
Antibody-based	94 (3.8)	41 (4.0)	53 (3.7)		
Antigen-based	2 (0.1)	1 (0.1)	1 (0.1)		
None	1,777 (71.8)	746 (72.6)	1,031 (71.3)		
**Symptoms (if yes to infection, n=** 687^**^(n_**male**_ **= 278, n**_**female**_ **= 409)) (n, %)**					
any symptom				**0.001**	2
Yes	578 (84.1)	218 (78,4)	360 (88.2)		
No	107 (15.6)	59 (21.1)	48 (11.7)		
bedridden				0.88	37
Yes	268 (39)	105 (37.8)	163 (41.5)		
No	382 (55.6)	152 (54.7)	230 (56.2)		
hospitalized	15 (2.1)	10 (3.6)	5 (1.2)	**0.035**	7
**Vaccination status (n,%)**					13
not vaccinated	513 (20.7)	195 (19,0)	318 (22.0)	0.097	
vaccinated with one dose	1,812 (73.2)	765 (74.7)	1,047 (72.9)		
vaccinated with two doses	136 (5.5)	64 (6.3)	72 (5.0)		
**Vaccine type among vaccinated, (n=1,948) n**_**male**_ **= 829, n**_**female**_ **= 1,119) (n, %)**				0.14	8
Comirnaty (BioNTech/Pfizer)	1,785 (91.6)	766 (92.4)	1,019 (91.1)		
Vaxzevria (AstraZeneca)	147 (7.5)	56 (6.8)	91 (8.1)		
Spikevax (Moderna Biotech)	7 (0.4)	1 (0.1)	6 (0.5)		
Others	1 (0.1)	1 (0.1)	0		
**Antibody status (n,)**					0
anti-S IgG positive	1,271 (51.4)	515 (50.1)	756 (52.3)	0.28	
Anti-N Ig positive	848 (34.3)	340 (31.3)	508 (35.1)	0.29	1
**pVNT (50% inhibition titer)**^**§**^, **Overall (n=848) (n**_**male**_ **= 340, n**_**female**_ **= 508)**					0
pVNT positive (≥16), n (%)	757 (89.3)	299 (87.9)	458 (90.2)	0.31	
Median (GMT)	695.9 (265)	674.9 (260.2)	708.5 (265.4)	0.19	
**By vaccination status**					5
***Non-vaccinated, (n=248) (n**_***male***_ **= 89, n**_***female***_ **= 159)***					
pVNT positive (≥16), n (%)	171 (69.0)	56 (62.9)	115 (72.3)	0.13	
Median (GMT)	24.5 (17.7)	24.4 (16.1)	24.5 (18.6)	0.57	
***Vaccinated, (n=595) (n**_***male***_ **= 251, n**_***female***_ **= 344)***					
pVNT positive (≥16), n (%)	584 (98.2)	243 (96.8)	341 (99.1)	**0.04**	
Median (GMT)	1,129.0 (820,4)	1,075.9 (697.7)	1,163.2 (923.4)	0.05	

* Significance was calculated using chi-square test for the categorical variables and Mann-Whitney U test for the continuous variables,

** 687 = 591 (PCR-positive) + 94 (antibody-positive) + 2 (antigen-positive),

§ Includes only subjects positive for anti-N Ig.

SD, standard deviation; IQR, interquartile range; S, spike protein; N, nucleocapsid protein; pVNT, pseudovirus based virus neutralization test; GMT, geometric mean titer.

### Seropositivity and previous infection

Independent of sex, the proportion of subjects positive for anti-S IgG antibody was 51.4% and positive for anti-N antibody 34.3% (*p* = 0.28 and 0.29, respectively) (Table 1). The proportion of anti-S positives was higher since there were participants who had been vaccinated prior to or as part of the mass immunization programme.

In order to assess the seroprevalence of SARS-CoV-2 antibodies before the mass vaccination, we characterized (as shown in Table 2) subjects with positive anti-N Ig antibodies (*n* = 848). We observed a significant association between anti-N Ig levels and age but no difference across sexes. The median concentration was three times higher among participants older than 60 years as compared to those below 40 years of age. Only 548 (64.6%) anti-N Ig positive subjects also reported a history of previous PCR- or antigen-confirmed SARS-CoV-2 infection. About one third of the remaining 296 subjects with no history of PCR- or antigen-confirmed SARS-CoV-2 infections reported to have found out about a previous infection based on an antibody test prior to the study, taking 205 participants (8.3% of the total study participants) by surprise. Participants reporting severe symptoms or a history of hospitalization had significantly higher anti-N Ig concentrations than participants without (*p* = 0.008 and 0.018, respectively). The mere presence or absence of symptoms showed no significant difference on the level of antibodies directed against N protein.

**Table 2:** Geometric mean (median) anti-N antibody concentrations across participant characteristics (n=848).

	***n***	**Geometric mean (median), COI**	*p*-value^**^	**Missing (n)**
**Participants**			0.25	0
male	340	35.1 (42.5)		
female	508	30.7 (37.4)		
**Age (years)**			**<0.0001**	0
<40	365	23.5 (24.8)		
40- <60	363	38.2 (44.5)		
>60	120	52.2 (68.3)		
**History of infection**			**0.034**	4
Yes (PCR, AG, AB)	639	35.1 (42.4)		
No	205	27.5 (35.3)		
**History of infection**			0.32	4
Yes (PCR, AG)	548	34.1 (41.5)		
No^*^	296	29.8 (37.4)		
**Symptoms (n**_**historyofinfection**_ **=639)**			0.52	2
Yes	550	36.1 (42.9)		
No	87	30.1 (36.2)		
**Non-mild symptoms (bed-bound), (n**_**historyofinfection**_ **=639)**			**0.008**	27
Yes	258	42.3 (46.0)		
No	354	31.5 (40.5)		
**Hospitalized (n**_**historyofinfection**_ **=639)**			**0.018**	5
Yes	15	88.8 (119.8)		
No	619	34.3 (41.6)		
**pVNT (50% inhibition titer)**^**§**^**, (n=248)** positive (≥ 16)	171	53.4 (40.6)	**<0.0001**	0
Negative (<16)	77	24.4 (19.4)		

* Including participants with a history of antibody positive,

** P-values were calculated based Mann-Whitney test,

§ Non-vaccinated convalescent individuals.

AB, antibody-based; AG, antigen-based; COI, coefficient of index. significant findings (values < 0.05) are in bold.

The majority of anti-N Ig positive subjects, 757 (89.3%), were also positive for neutralizing antibodies. However, a significant proportion had already received at least one dose of SARS-CoV-2 vaccines prior to our study impeding the interpretation of infection-induced neutralization activity. Compared to 98.2% of previously infected plus vaccinated individuals only 69.0% of the participants with previous infection without any history of vaccination were positive for neutralizing antibodies. The median 50% inhibition titer was 46 fold higher in the vaccinated plus infected group as compared to the non-vaccinated convalescent group (*p* < 0.0001). Concentrating on the group with a history of infection but no vaccination (*n* = 248), we observed a significant association between anti-N Ig level and positivity for neutralizing antibodies.

### Undocumented SARS-CoV-2 infections and standardized seroprevalence for the general adult population of Schwaz

Undocumented infection was defined as being anti-N Ig positive despite reporting to have had no known SARS-CoV-2 infection in the past. After excluding 548 persons who explicitely reported to have had prior PCR- or antigen-based infection, we found the crude seroprevalence of undocumented infections among the study population reporting no history of infection to be 15.8% (95% CI: 14.2–17.6). Supplementary Figure 2 shows the quantitative distribution of anti-N Ig values across age.

Since the age and sex distribution of the study participants showed obvious deviation from the official distribution of the total population in Schwaz (Supplementary Figure 1), we estimated the age and sex standardized prevalence (95% CI) of undocumented infections as shown in Table 3. We initially estimated the number of individuals in the general population with undocumented infections by projecting the age-specific crude seroprevalence from the study population to the total population. We then determined the overall prevalence of undocumented infections across age categories and sex. The sum of the prevalences across these age strata (15.0%, 95% CI: 13.3–16.7) was the overall age standardized prevalence among adults in Schwaz. This translated into 9,228 (95% CI: 8,150–10,296) undocumented infections out of 61,576 adults officially never having a previous SARS-CoV-2 infection (Table 4). Taking 7,320 subjects officially reported to have had confirmed SARS-CoV-2 at the time of data collection (information obtained from AGES), the true total number of infections in the adult population of the district of Schwaz by March 2021 was estimated at 7,320 + 9,228 = 16,548 (95% CI: 15,494 – 17,616) which translates into an overall pre-mass vaccination SARS-CoV-2 prevalence of 24% (95% CI: 22.5–25.6) and a proportion of undocumented infections of 55.8% (95% CI: 52.7–58.5) in adults at that time.

**Table 3:** Characterizing study population and source population without a known history of officially reported SARS-CoV-2 infection.

**Age group**	**Sex**	**Participants reporting no history of infection**	**Anti N positive participants**	Age–specific *crude* prevalence, % (95% CI)^*^	No. of people in reference population with no report of previous infection^**^	**(Expected number (n) of unreported cases in the reference population)^§^**	**Expected proportion (%, 95% CI) of unreported cases in the reference population]^§§^**	**% Reference population out of total**
<40	Female	480	78	16.3 (13.1-19.9)	10,347	1,681	2.73 (2.60-2-86)	16.8%
<40	Male	316	50	15.8 (12.0-20.3)	10,708	1,694	2.75 (2.62-2.88)	17.4%
40- <60	Female	469	80	17.1 (13.8-20.8)	10,980	1,873	3.04 (2.91-3.18)	17.8%
40- <60	Male	345	58	16.8 (13.2-21.2)	11,045	1,857	3.02 (2.88-3.15)	17.9%
60+	Female	135	15	11.1 (6.35-17.7)	9,940	1,104	1.79 (1.69-1.90)	16.1%
60+	Male	126	15	11.9 (6.82-18.9)	8,556	1,019	1.65 (1.56-1.76)	13.9%
**Total**		**1,871**	**296**	**15.8** **(14.2-17.6)**	**61,576**	**9,228**	**15.0%**	

* Proportion of anti-N positives across age and sex strata,

** Based on daily reports of SARS-CoV-2 infection in Schwaz since the beginning of the pandemic (data obtained from AGES),

§ Calculated as: $\frac{age\;specific\;crude\;prevalence\;X\;number\;of\;people\;in\;the\;reference\;population\;with\;no\;report\;of\;previous\;infection}{100}$,

§§ Calculated as: $\left(\frac{Expected\;number\;\left(n\right)\;of\;unreported\;cases\;in\;the\;reference\;population}{61576}\right)X\;100$%. all 95% confidence intervals (CI) calculated based on Clopper-Pearson exact method.

**Table 4:** Projected undocumented and overall point prevalence of SARS-SoV-2 infection in the district of Schwaz just before the mass immunization campaign.

	**Point estimate**	**95% confidence interval**	
		**lower bound**	**upper bound**
**Estimated no. of cases in Schwaz**			
Expected number of unreported cases in the reference population	9,228	8,159	10,296
Previously documented*	7,320		
Documented and undocumented cases	16,548	15,479	17,616
% previously undocumented	55.87%	52.7%	58.5%
% of reference population including previously documented cases**	24.0%	22.5%	25.6%

*Data from AGES, **Calculated as: $\left(\frac{16548}{68896}\right)X\;100$%, all 95% confidence intervals (CI) calculated based on Clopper-Pearson exact method.

In a sensitivity analysis that employed the Rogan-Gladen correction for an imperfect diagnostic test (Supplementary Table 1), the results were similar to the principal analysis, as expected given the sensitivity and specificity of the anti-N Ig assay close to 100%. In specific, in participants without a known history of SARS-CoV-2 infection, the age- and sex-standardized seroprevalence was 14.9% (95% CI: 13.2–16.6%) and the expected number of unreported cases in the reference population was 9,169 (95% CI: 8,103–10,233) after correction.

## Discussion

At the time of our data collection, the mass vaccination had been going on for about one week. In order to avoid a potential bias caused by anti-S IgG seroconversion following vaccination, we chose to use anti-N Ig in estimating the seroprevalence. This helps to doubtlessly exclude the effect of vaccination following SARS-CoV-2 infection, since earliest seroconversion has been described to occur within days post vaccination (16–20). The demonstrably good performance of the Roche anti-N immunoglobulin assay underscores the validity of our approach (21–23). Moreover, the assay proved a persistently high sensitivity even months after a confirmed infection (23).

With 7,320 officially documented cases above the age of 18 at the time of the study, the official SARS-CoV-2 prevalence would be estimated to 10.6% (95% CI: 10.4–10.9) at that time. The fact that 24% of the study participants reported to have had PCR- or antigen-confirmed infection led to the reasonable suspicion that our convenience sampling may have resulted in a selection bias. A seroprevalence of 34.3% based on anti-N positivity is thus likely to be an overestimation. Consequently we opted to include, in the main analysis, only subjects who reported no known history of infection (*n* = 1,871) and to estimate age standardized cumulative incidence of unreported infections in the general adult population of the district of Schwaz (*n* = 61,576). Using data obtained from official statistics in Austria (14), we accounted for disproportional age and sex distribution of the study population by conducting direct age and sex standardization. Our finding of standardized seroprevalence of 15.0% translates into previously undocumented 9,228 cases (95% CI: 8,159–10,296) in the general adult population of Schwaz. Adding this to 7,320 (10.6%) subjects officially reported to have had SARS-CoV-2 at the time of data collection (data from AGES), the overall prevalence of SARS-CoV-2 infection at the start of the mass vaccination among adults added up to 24% (95% CI: 22.5–25.6). With a vaccination coverage of 10% among the adult population of Schwaz prior to the mass immunization campaign (6), our result implies that a maximum of one third of the adult popuation had at least one SARS-CoV-2 specific immunological event.

### Previous studies

Comprehensive meta-analysis studies indicate that a plethora of seroprevalence studies has been conducted across the globe since early on in the pandemic. A wide range of cumulative incidence has been reported depending on the population studied, the sample size used, the serological method applied, the time of the study since the start of the pandemic or whether or not vaccination status was considered (24, 25), making direct comparison of estimates very challenging.

Based on a review on population-based studies in Europe until September 2020, for example, the seroprevalence ranged from as low as 0.42% in some studies to as high as 23.3% in a highly affected region of Lombardy, Italy following the first wave of infection (24). Another study conducted in Ischgl, a once-a-corona-hotspot ski resort in western Austria in April 2020, found an even higher seroprevalence of 42% (26). A nation-wide study in Austria, conducted in Autumn of 2020 using a representative sample of the Austrian population, found a seroprevalence of 4.7% (95% CI: 3.8–5.6) and reported that the estimate for western Austria was higher (5.7%, 95% CI: 4.1–7.4) (27).

Our study was conducted at the end of the second infection wave, hence the higher seroprevalence highlighting the temporal continuum of the rising infection numbers. A follow-up nationwide survey conducted between October 2020 and January 2021 and global epidemiological data on seroprevalence over time also corroborate this temporal trend (25, 28).

Regarding antibody concentration and age, our findings are in accord with several previous works showing higher antibody titres in older adult populations, presumably owing to the fact that age is a known risk factor for severe disease (29–32). Since the very beginning of the pandemic, even before vaccines were available, several studies reported higher levels of both binding and neutralizing antibodies correlating with disease severity, which in turn has been shown to correlate with age among other factors (33–37).

Similarly, a superiority of the concentration and quality of antibodies generated by vaccinees with a history of infection compared to immuno-naïve vaccinees has also been reported previously, even following a single dose (18, 38–45). The robustness of this finding is strengthened by the observation that not only humoral response but also cellular immunity was generally superior in the non-naïve group (40, 43, 45).

### Seroprevalence post second infection wave

Since seroprevalence surveys conducted after 2021 paralleled the vaccination rollout, interpretation of data has been a challenge unless non-vaccine-induced antibodies like the nucleocapsid protein antibodies are targeted. Similar to ours, several other studies investigated seroprevalence based on anti-N Ig in the post-vaccine era. Studies targeting health care workers or patients or residents of nursing homes found a much higher seroprevalence than ours owing to the high-risk target population (46–48). For instance, a prospective study from England, which examined the prevalence of SARS-CoV-2 among both residents and staff in long-term care facilities over a period of March through the beginning of May 2021 found a prevalence of anti-N Ig positivity of 34.6% among the residents and 26.1% among staff (47).

Blood donors, on the other hand, make up a comparable group to our study population. Contemporaneous to ours, another survey assessed seroprevalence among Tyrolean blood donors targeting anti-N antibodies. Siller et al. found seropositivity (95% CI) of 14.0% (13.0–15.1) among donor samples collected in March 2021 (49), a finding notably lower than ours (24%) in the district of Schwaz. Several factors may be accountable for this: First, the two studies used different serological platforms—chemiluminescent microparticle immunoassay from Abbott vs. Electrochemiluminescence immunoassay from Roche. These two assays were shown to have a significant gap in sensitivity of detecting anti-N antibodies particularly later in the post-infection period in favor of the Roche assay (40): Six months post confirmed SARS-CoV-2 infection, the Roche assay had a sensitivity of 94,3% (95% CI: 84.3–98.8) in detecting anti-N antibodies whereas for the Abbott system it was only at 45.3 % (95% CI: 31.6–59.6%) (39). Second, reports from official data indicated that the district of Schwaz had higher incidence rates (49, 50) in the second infection wave as compared to the rest of the Tyrolean districts, further accounting for the discrepancy.

Common to the majority of previously published seroprevalence data, as underpinned by a large global meta-analysis, is that the estimates based on serological approach are clearly higher than the officially reported ones with values ranging from as low as 1.5 times to as high as 10 times the reported cumulative incidence (51). With 55.8% of seropositive subjects having never been registered officially, our study underpins this notion.

### Strengths and limitations

Beyond the large sample size, the availability of data on daily infections in the study district across age and sex since the start of the pandemic, as well as the official census data that enabled a direct age standardization and projection of estimates to the general population, is one strength of the study. The main serological assay we used to determine seroprevalence has a high sensitivity and specificity (22, 23).

A major limitation of this study is the convenience-sampling approach in which individuals who were willing to participate may be significantly different from those who were not attracted by this approach. This might have resulted in selection bias toward higher prevalence. We aimed to balance this effect by concentrating on subjects reporting no known history of previous infection and by coupling the unreported infections to the officially registered cases. A further limitation is that our data concentrated only on the adult population as we lacked ethical clearance to include minors.

## Conclusion

Our conclusions are twofold. First, by accounting for the undocumented cases through serological approach, our study confirmed once again that officially reported data on infection status markedly underestimate the true prevalence. Second, adding our finding to the vaccine coverage of 10% among the adults population shortly before the mass immunization campaign (6), our result implies that at least two-thirds of the adult population was immuno-naïve and susceptible to the circulating threat as this unique campaign started.

## Data availability statement

The original contributions presented in the study are included in the article/Supplementary material, further inquiries can be directed to the corresponding author/s.

## Ethics statement

The studies involving human participants were reviewed and approved by Ethics Committee of the Medical University of Innsbruck (EK Nr:1093/2021). The patients/participants provided their written informed consent to participate in this study.

## Author contributions

Conceptualization: DL and WB. Data and sample acquisition: HS, DB, BF, TH, CO, MS, and WB. Laboratory work: JK, WB, AR, and LR. Data cleaning: TH and WB. Formal data analysis: PW, WB, and HW. Manuscript drafting: WB and PW. All authors have read, critically reviewed, and agreed to this version of the manuscript.

## Conflict of interest

DB declares to hold stocks of Pfizer. The remaining authors declare that the research was conducted in the absence of any commercial or financial relationships that could be construed as a potential conflict of interest.

## Publisher's note

All claims expressed in this article are solely those of the authors and do not necessarily represent those of their affiliated organizations, or those of the publisher, the editors and the reviewers. Any product that may be evaluated in this article, or claim that may be made by its manufacturer, is not guaranteed or endorsed by the publisher.

## Supplementary material

Supplementary Figure 1 Age structure of the study population as compared to the official age structure in Schwaz (source population) among men **(A)** and women **(B)** [Source population data from Statisik Austria (14)].

Supplementary Figure 2 Spearman‘s correlation coefficient (r) and 95% CI between anti N Ig and age of study participants with no reports of known prior infection (*n* = 1871) **(A)** and the whole study participants (*n* = 2472) **(B)**. Dotted horizontal lines represent the cutoff values as recommended by the manufacturer. COI, coefficient of index; N, nucleocapsid; Ig, Immunoglobulin.

Supplementary Table 1 Seroprevalence of unreported SARS-CoV-2 infection after Rogan-Gladen correction.
