# Supplementary figures and images for: Seroprevalence of SARS-CoV-2 infection in the Tyrolean district of Schwaz at the time of the rapid mass vaccination in March 2021 following B.1.351-variant outbreak
Source: Front Public Health. 2022 Sep 9;10:989337. doi: 10.3389/fpubh.2022.989337 (PMC9500479; doi:10.3389/fpubh.2022.989337)

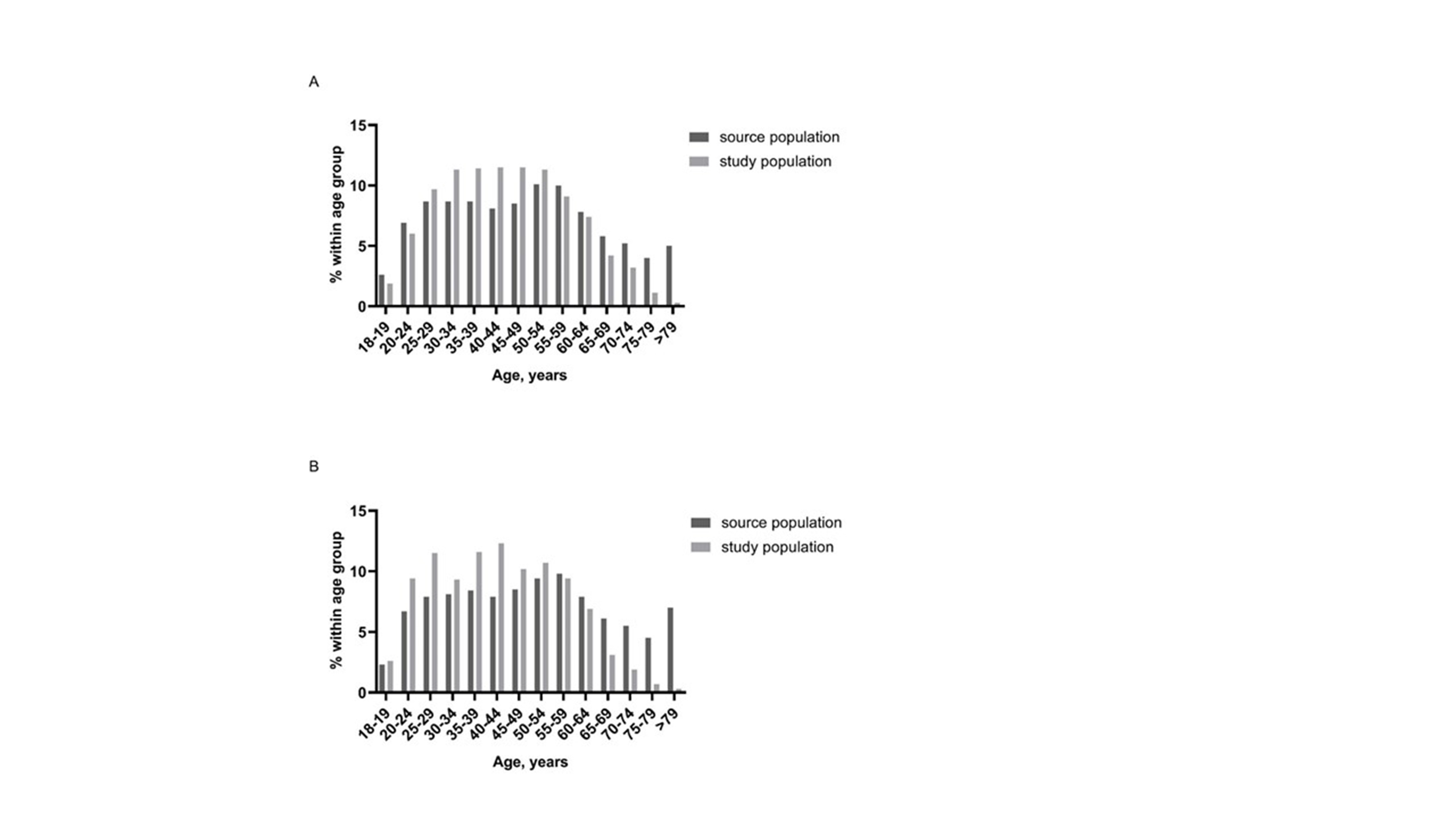

Supplement: Supplementary Figure 1 — Age structure of the study population as compared to the official age structure in Schwaz (source population) among men (A) and women (B) [Source population data from Statisik Austria (14)]. [file Image_1.jpg]

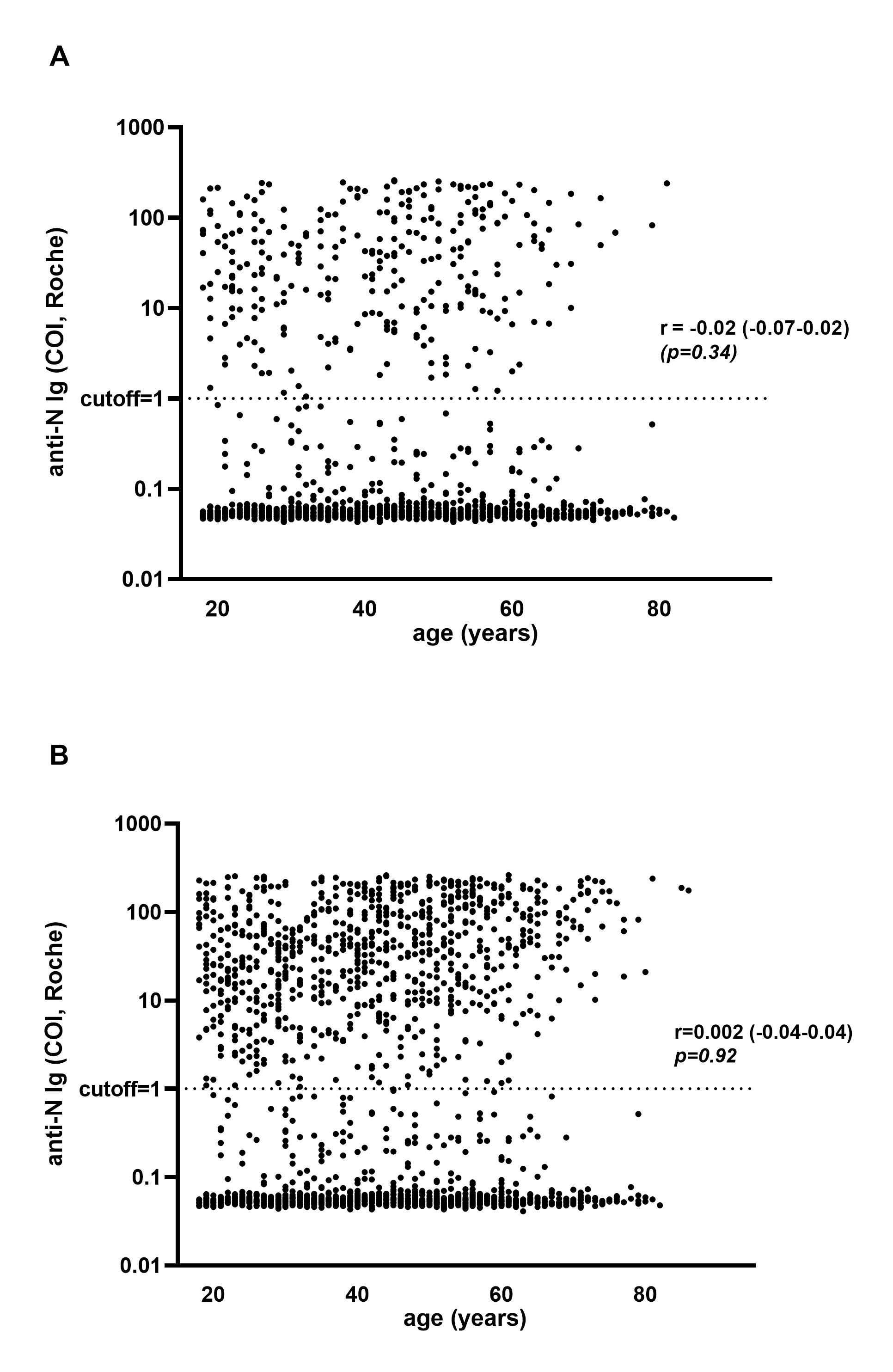

Supplement: Supplementary Figure 2 — Spearman‘s correlation coefficient (r) and 95% CI between anti N Ig and age of study participants with no reports of known prior infection (n = 1871) (A) and the whole study participants (n = 2472) (B). Dotted horizontal lines represent the cutoff values as recommended by the manufacturer. COI, coefficient of index; N, nucleocapsid; Ig, Immunoglobulin. [file Image_2.jpg]
