# Supplementary material for: Seroprevalence of SARS-CoV-2 infection in the Tyrolean district of Schwaz at the time of the rapid mass vaccination in March 2021 following B.1.351-variant outbreak
Source: Front Public Health. 2022 Sep 9;10:989337. doi: 10.3389/fpubh.2022.989337 (PMC9500479; doi:10.3389/fpubh.2022.989337)
Supplement: Supplementary Table 1 — Seroprevalence of unreported SARS-CoV-2 infection after Rogan-Gladen correction. [file Table_1.docx]

| **Age group** | **Sex** | **Age-specific crude prevalence*** | **Rogan-Gladen corrected**** | **% reference population of total** | **Expected proportion of unreported cases (source population)^§^** | **Source population with no reports of infection^§§^** | **Expected number (n) of unreported cases in the reference population‡** |
| --- | --- | --- | --- | --- | --- | --- | --- |
| <40 | Female | 16.3 | 16.2 | 16.8 | 2.72 | 10,347 | 1,675 |
| <40 | Male | 15.8 | 15.7 | 17.4 | 2.73 | 10,708 | 1,680 |
| 40-<60 | Female | 17.1 | 17.0 | 17.8 | 3.02 | 10,980 | 1,864 |
| 40-<60 | Male | 16.8 | 16.7 | 17.9 | 2.99 | 11,045 | 1,842 |
| 60+ | Female | 11.1 | 11.0 | 16.1 | 1.77 | 9,940 | 1,095 |
| 60+ | Male | 11.9 | 11.8 | 13.9 | 1.64 | 8,556 | 1,011 |
| Total | |  | |  | 14.9% |  | 9,167 |

Supplementary table 1. Seroprevalence of unreported SARS-CoV-2 infection after Rogan-Gladen correction

*Proportion of anti-N positives across age and sex strata (shown in table 3),

**Sensitivity and specificity (Roche) =0.995 (95% CI 0.97-1) and 0.998 (0.997-0.999), respectively (according to the manufacturer),

**Calculated as: $\frac{\left[ age specific crude prevalence+\left( Specificity-1 \right) \right]}{Specificity+(Sensitivity-1)}$ ,

**^§^**Calculated as: $\left( \frac{Expected number \left( n \right) of unreported cases in the reference population}{61576} \right)X 100$%,

**^§§^**Based on daily reports of SARS-CoV-2 infection in Schwaz since the beginning of the pandemic (data obtained from AGES),

‡Calculated as: $\frac{age specific crude prevalence X number of people in the reference population with no report of previous infection}{100}$.
